# Plant–Microbe Interaction 2017—The Good, the Bad and the Diverse

**DOI:** 10.3390/ijms19051374

**Published:** 2018-05-05

**Authors:** Jan Schirawski, Michael H. Perlin

**Affiliations:** 1 Microbial Genetics, Institute of Applied Microbiology, RWTH Aachen University, Worringerweg 1, 52074 Aachen, Germany; 2 Department of Biology, Program on Disease Resistance, University of Louisville, Louisville, KY 40292, USA; michael.perlin@louisville.edu

**Keywords:** plant–microbe interactions, microbiome, transcriptome, effectors, comparative methods, *Streptomyces*, plant growth-promoting bacteria, phytoremediation, rhodopsins

## Abstract

Of the many ways that plants interact with microbes, three aspects are highlighted in this issue: interactions where the plant benefits from the microbes, interactions where the plant suffers, and interactions where the plant serves as habitat for microbial communities. In this editorial, the fourteen articles published in the Special Issue Plant–Microbe Interaction 2017 are summarized and discussed as part of the global picture of the current understanding of plant-microbe interactions.

## 1. Introduction

Often not visible to the naked eye, interactions between plants and microorganisms occur in many different ways and on many different levels. Virtually all organs of the plant interact with microorganisms at a certain stage of their life, and this interaction is not necessarily negative for the plant. Indeed, there are plenty of interactions where the plant benefits either through direct or through indirect effects of the associated microbes. In these interactions, plants serve as sheltered habitats for the microorganisms that may colonize apoplastic spaces, plant surface areas or areas adjacent to the plant surface, e.g., the rhizosoil, the soil in the vicinity of roots. In addition to a sheltered habitat and a future source of nutrients that are liberated upon plant death, many plants release compounds that attract and feed the associated microbes. The associated microbes may in turn secrete compounds that favor plant growth, they may make the plant more resistant to abiotic or biotic stress, or they may defend the plant against more malignant microbes.

With the development of techniques to identify and quantify the microbial diversity associated with plants, we begin to grasp the immensity of the interactions to which plants are exposed. Complete microbiomes can be evaluated that are associated with different parts of the plant, and microbes can be found wherever they are looked for. Since functional effects of these multidimensional interactions are difficult to disentangle, most researchers stick to easier tractable and less complex interactions that can be experimentally tackled. This holds also true for researchers studying plant–microbe interactions that are clearly negative for the plant and result in the development of plant disease symptoms. Research on these negative interactions focuses at identifying microbial and plant factors necessary for establishment of the disease and elucidating their molecular function.

The sum of all these research endeavors will lead to an increased, detailed and more and more complex understanding of the multidimensional interactions that plants keep with microbes. In this issue, three aspects of plant–microbe interaction are highlighted: the good, the bad and the diverse. In twelve original research articles and two reviews, we will learn about different interactions where the plant profits from the interaction with the microbes, where it suffers, and where it serves as a habitat for a microbial community. Below, we will summarize the most interesting highlights.

## 2. The Good: Soil Microbes Positively Affecting Plant Growth

Of the microorganisms colonizing the rhizosoil, *Streptomyces* species are special. They grow filamentously and can colonize not only soil but also roots and aerial parts of the plants; they are active producers of antibiotics and can save the plant from attack by more dangerous bacteria; and they produce volatile organic compounds that give rise to the typical fragrance of fresh forest soil. As such, they qualify as biocontrol agents in several cropping systems, and strains serving as antagonists of various plant pathogens can be identified. The versatile *Streptomyces* species also have plant growth-promoting abilities and can be used as biofertilizers. Because of their ability to form spores and survive adverse conditions in the soil, they are also more competitive than other microbes. In addition, they produce various lytic enzymes that can break down insoluble organic polymers and generate nutrients that can be used by plants. These fascinating aspects of *Streptomyces* species are nicely summarized in the review by Vurukonda et al. [1].

Plant growth-promoting bacteria can also be used in phytoremediation of metal-contaminated soils. Montalbán et al. show that exposing *Helianthus tuberosus*, a high biomass crop used for bio-ethanol production, to particular plant growth-promoting bacteria that were isolated from plants growing on a metal-contaminated soil increased the ability of the plant to sustain elevated concentrations of cadmium and zinc [2]. The bacteria were shown to grow endophytically in the root and resulted in a significantly increased cadmium uptake into the plant. In presence of the bacteria, the plant showed a decrease of metal-induced stress and an improved growth. Thus, these plant growth-promoting bacteria can help both in phytoremediation and in sustainable biomass production [2].

Plant growth-promoting bacteria can induce drought and salt tolerance. Two articles study the effect of plant growth-promoting bacteria on perennial ryegrass (*Lolium perenne*), an important cool-season perennial grass species for pasture, forage and turf with high yield and good turf quality such as a dense root system, superior tillering, and regeneration ability. Unfortunately, this popular grass species is not very tolerant to drought or to high salinity. Su et al. show that the beneficial soil bacterium *Bacillus amyloliquefaciens* GB03 together with a water-retaining agent consisting of super absorbent hydrogels used for soil erosion control can significantly improve the drought resistance of perennial ryegrass; this was true even compared to application of single components that already significantly improve drought resistance of the plant relative to control [3]. He et al. used a novel bacterium isolated from a C4 perennial succulent xerohalophyte shrub with excellent drought and salt tolerance to significantly increase both the growth and salt tolerance of perennial ryegrass [4]. In addition, they sequenced the bacterial genome and identified several genes putatively involved in plant growth-promoting traits and abiotic stress tolerance [4].

Zhang et al. studied the positive role of the arbuscular mycorrhizal fungus *Rhizophagus irregularis* CD1 on plant growth promotion and the *Verticillium* wilt resistance of cotton [5]. They determined the symbiotic efficiency of 17 cotton varieties to *R. irregularis*. The best one, Lumian 1, was used for a two-year field trial. Presence of the mycorrhizal fungus significantly increased plant growth and plant disease resistance against *Verticillium dahliae* wilt. While the negative effect on *V. dahliae* colonization could be due to mycorrhiza-induced resistance, the authors show that growth of *R. irregularis* may directly inhibit growth of *V. dahliae* by releasing as yet unknown volatiles [5].

Thus, microorganisms can be used to positively change growth capacities of plants and to make them more resistant against biotic and abiotic stresses like draught and salt, stresses that will likely occur much more often with progressing climate change. We will need to increase our knowledge about how these systems function in order to tackle the challenges of the future and ensure plant fitness under increasingly adverse conditions.

## 3. The Bad: Elucidating Mechanistic Strategies of Plant Pathogens

Of the plant-pathogenic microorganisms, fungi are an enormous threat to plant health. While a lot of plant-pathogenic fungi are highly host-specific, host switching events are often at the base of emerging fungal diseases. Therefore, the elucidation of host-specificity factors that allow fungal proliferation and disease formation on particular host plants is one of the hot research topics in plant pathology. In the review by Borah et al., comparative methods for the molecular determination of host-specificity factors are discussed [6]. It turns out that the elucidation of host-specificity factors requires several successive steps, for each of which several comparative methods exist. In most cases, comparison of molecular characteristics of different host-specific strains or species resulted at best in a list of target genes potentially involved in host specific virulence that await verification and functional validation. The authors indicate that intelligent combination of classical genetics, genomics, and transcriptomics covering both the pathogen and the host may lead to host-specificity factor identification and a mechanistic understanding of host specificity [6].

Biotrophic plant-pathogenic fungi live in close intimacy with the plant because they feed on living plant tissue and have to subvert the defense systems of the plant. One of their strategies for survival in the hostile plant tissue environment is the secretion of effector proteins that interact with plant proteins to the advantage of the pathogen. In their contribution, Kuppireddy et al. have analyzed the genome of *Microbotryum lychnidis-dioicae*, a biotrophic fungus causing anther smut on a common weed, *Silene latifolia*, to identify putative effector proteins [7]. Out of 50 identified putative effectors, they showed for four that they are indeed secreted proteins. Interaction analysis revealed a plant protein with homology to a protein involved in pollen germination. Considering that *M. lychnis-dioicae* forms spores exclusively in anthers, the places of pollen generation, this interaction may lead to tantalizing insights into the interaction of the fungus with its host tissue [7]. Gao et al. also identified effectors but in a different pathosystem [8]. They generated and analyzed the transcriptome of *Fusarium proliferatum*, the causal agent of a destructive tomato disease during which dark brown necrotic spots appear on leaves and stems that grow and cause stems to soften and wilt, often leading to death of the entire tomato plant. In the absence of a published genome sequence, they resorted to de-novo assembly of the sequenced transcriptome and analyzed gene expression to identify 184 putative effector candidates, most displaying elevated expression during plant colonization [8]. In a related study, Wang et al. analyzed the transcriptome of Kiwifruit in response to infection by the bacterial canker pathogen *Pseudomonas syringae* pv. *actinidiae* (Psa) [9]. Gene expression analysis of the infected kiwifruit plant revealed upregulation of several genes. These included key genes for defense compound (terpene) biosynthesis and the generation of secondary metabolites, genes involved in plant immunity (pathogen-associated molecular pattern-induced immunity and effector-triggered immunity), as well as a change in expression of metabolic processes that may all have a role in suppressing spread of Psa [9].

Adam et al. analyzed a completely different aspect in the interaction of fungal pathogens with plants [10]. They found that phytopathogenic and phyto-associated ascomycetes contain rhodopsin-encoding genes. The rice plant pathogen *Fusarium fujikuroi* contains two different rhodopsins, CarO and OpsA. While CarO was previously shown to be a light-driven proton pump, here the authors show that CarO is positively regulated by presence of indole-3-acetic acid and of sodium acetate. Intriguingly, they showed that deletion of the CarO-encoding gene from the genome of *F. fujikuroi* resulted in a hypervirulent strain with more severe bakanae symptoms than the reference strain, indicating that CarO has a role in attenuating the disease potential of the fungus [10]. Thus, although our knowledge on how plant pathogens infect host plants and on how the plants react to pathogen attack steadily increases, much remains still unknown. As the last example shows, research often reveals unexpected results that stimulate further research and necessitate an adjustment of the current plant–pathogen interaction models.

## 4. The Diverse: Microbiomes of Seeds and Roots

Plants are covered by microbes: some of them cause disease, some have a positive influence on plant growth, and some microbes may just be there with an as-yet undiscovered role in microbial ecology. Roots are surrounded by a thick layer of associated microbes in the rhizosoil, and not even seeds are sterile. Microbes associated with seeds can have a profound influence on plant development, since they are present upon seed germination and can affect plant ecology, health and productivity. The seed microbiome comprises both endophytic microbes as well as microbes present on the seed surface. Chen et al. investigated whether there was a core microbiome associated with the seeds of a medicinal plant, *Salvia miltiorrhiza*, used for traditional treatment of coronary and cerebrovascular diseases [11]. The plant is also known to contain active secondary metabolites, such as salvianolic acid and tanshinone, a diterpenoid quinone. They collected seeds from different geographic cultivation areas and determined the total seed-associated microbiomes. They compared the seed microbiomes of the different locations and also between those of *S. miltiorrhiza* and other commonly cultivated crop plants. The authors found a clear overlap of microbial taxa associated with seeds of *S. miltiorrhiza*. In contrast, the overlap in microbiomes of seeds of different plants was limited to a few microbial species. Interestingly, the authors found that in the core bacterial microbiome, genes for secondary metabolism were overrepresented, including genes encoding prenyltransferases, terpenoid backbone biosynthesis enzymes, as well as enzymes for degradation of limonene, pinene and geraniol. This suggests a possible contribution of the microbiome to the secondary metabolite profile of medicinal plants [11].

In a parallel study, Sánchez-López et al. investigated whether seed-associated microbes could be transmitted vertically through several generations [12]. They investigated seeds of *Crotalaria pumila*, a pioneer plant in metal-contaminated soils. The most prominent community member of the seed-associated microbiome of *C. pumila* over three generations was a *Methylobacterium* sp. Cp3 [12]. The authors could show that root inoculation of flowering plants with strain Cp3 led to the occurrence of Cp3 in the seeds. Using tagged strains, the authors followed the bacteria colonizing the root cortical cells and the xylem vessels in the stem of *C. pumila* under metal stress. They present evidence consistent with a positive role of strain Cp3 for seed germination and seedling development [12]. This shows that the seed microbiome may contribute significantly to the general fitness of the plant and may even be involved in adaptation of the plant to adverse living conditions like metal-contaminated soils.

That roots are associated with microbes is common knowledge. What is less well-known is that the microbes in the rhizospheres of different plants affect each other to the advantage of the plants. Li et al. compared the root-associated microbiomes of maize and peanut either grown in monoculture or in intercropping [13]. It turns out that intercropping resulted in a higher microbial diversity with a higher accumulation of beneficial bacteria in the soil that led to increased levels of soil-available nutrients and an increase in plant biomass [13]. While this study showed the advantage of intercropping, in many areas, plant monocultures are cultivated successively on the same field. Plantations of tea (*Camella sinensis*) can be grown for over 30 years on the same field. Compared to new tea fields planted only two years ago, the older tea fields endure poor growth, chlorosis, wilting, and ratooning problems. Arafat et al. have compared the rhizosoils of young and old tea monoculture soils by measuring the bacterial diversity, the physicochemical properties of the soil and the content of plant exudates (metabolites leaking from the plants into the soil via the roots) [14]. While the physicochemical properties of the soils were nearly identical, the authors noticed an enhancement of catechin-containing compounds and a lowering of the pH of the soils with continued tea monoculture, which affected microbial distribution patterns. The authors suspect that plant exudates influence the bacterial community of the associated soil, which might lead to the described problems in yield reduction [14].

## 5. Conclusions

In this issue of Plant–Microbe Interactions 2017, two interesting reviews and twelve research articles highlight three aspects of plant–microbe interaction. Studying the beneficial interactions can enable us to increase plant fitness without the application of plant protection chemicals. These discoveries have a direct influence on agricultural practices, which justifies research to mechanistically understand how the plant growth-promoting microorganisms exert their beneficial effects. Of at least equal importance is understanding how plant pathogens can cause disease. Whereas effector proteins are suspected to be responsible for manipulating the plant’s defense systems and the plant’s metabolism to the advantage of the pathogen, the multitude of newly discovered effectors leaves a big gap in the understanding of how the particular effector proteins function. In addition, other genes of the pathogen might also affect their plant infection capacities, putatively opening the door to the development of novel plant protection strategies. Finally, the diversity of microbial communities is shown here to be not only responsible for ecosystem stability but have multiple positive effects on plant growth, disease resistance or tolerance towards abiotic stresses. Further research in this field is needed to finally understand the network interactions within microbial communities living on, in or near plants and influencing plant fitness on several levels. Therefore, in spite of new insights, research on plant–microbe interaction will continue to provide unexpected discoveries that help in understanding the microbial interaction network of plants. This in turn will empower us to optimize plant cultivation and provide food for an ever-growing population.
